# Two-row, three-row or powered circular stapler, which to choose when performing colorectal anastomosis? A systematic review and meta-analysis

**DOI:** 10.1007/s00384-024-04625-8

**Published:** 2024-04-12

**Authors:** José Martín-Arévalo, Vicente Pla-Martí, Dixie Huntley, Stephanie García-Botello, Leticia Pérez-Santiago, A. Izquierdo-Moreno, LP. Garzón-Hernández, M. Garcés-Albir, A. Espí-Macías, David Moro-Valdezate

**Affiliations:** 1 Colorectal Surgery Unit, Department of General and Digestive Surgery, Biomedical Research Institute INCLIVA, Hospital Clínico Universitario, Av. Blasco Ibáñez, 17. 46010, Valencia, Spain; 2 Department of Surgery, University of Valencia, Valencia, Spain; 3 Colorectal Surgery Unit, Department of General and Digestive Surgery, Instituto Valenciano de Oncología, Valencia, Spain; 4 Department of Anatomy, University of Valencia, Valencia, Spain

**Keywords:** Two-row manual circular stapler, Three-row manual circular stapler, Powered circular stapler, Anastomotic leak, Anastomotic bleed, Postoperative anastomotic complications

## Abstract

**Purpose:**

Three types of circular staplers can be used to perform a colorectal anastomosis: two-row (MCS), three-row (TRCS) and powered (PCS) devices. The objective of this meta-analysis has been to provide the existing evidence on which of these circular staplers would have a lower risk of presenting a leak (AL) and/or anastomotic bleeding (AB).

**Methods:**

An in-depth search was carried out in the electronic bibliographic databases Embase, PubMed and SCOPUS. Observational studies were included, since randomized clinical trials comparing circular staplers were not found.

**Results:**

In the case of AL, seven studies met the inclusion criteria in the PCS group and four in the TRCS group. In the case of AB, only four studies could be included in the analysis in the PCS group. The AL OR reported for PCS was 0.402 (95%-confidence interval (95%-CI): 0.266–0.608) and for AB: 0.2 (95% CI: 0.08–0.52). The OR obtained for AL in TRCS was 0.446 (95%-CI: 0.217 to 0.916). Risk difference for AL in PCS was − 0.06 (95% CI: − 0.07 to − 0.04) and in TRCS was − 0.04 (95%-CI: − 0.08 to − 0.01). Subgroup analysis did not report significant differences between groups. On the other hand, the AB OR obtained for PCS was 0.2 (95% CI: 0.08–0.52). In this case, no significant differences were observed in subgroup analysis.

**Conclusion:**

PCS presented a significantly lower risk of leakage and anastomotic bleeding while TRCS only demonstrated a risk reduction in AL. Risk difference of AL was superior in the PCS than in TRCS.

## Introduction

Anastomotic leakage following colorectal resection is the most feared complication for surgeons, affecting up to 36.3% of colorectal anastomosis [1].

Any colorectal surgeon should aim for an incidence of zero anastomotic leakages. However, this objective is likely impossible because the occurrence of anastomotic leakage depends on various factors involving non-modifiable risk factors such as patient characteristics and comorbidities (age, gender, ASA score, diabetes, tobacco use, corticosteroid use, administration of neoadjuvant treatments), as well as histopathological factors related to the disease that required the intervention [2–4]. Potentially modifiable risk factors to reduce the rate of this complication have been associated with optimizing preoperative albumin and haemoglobin levels, normalizing blood glucose levels, using minimally invasive surgical techniques, precise surgical timing [3], high ligation of the inferior mesenteric artery [5] and the use of a refined surgical technique [6].

The technique for performing a colorectal anastomosis should always meet the fundamental requirements of ensuring adequate vascularization, apposition of the intestinal ends to the anastomosis without tension and an appropriate and airtight intestinal lumen. Colorectal anastomoses can be performed manually or mechanically, with no clear evidence demonstrating the superiority of one technique over the other [7, 8].

Currently, most of colorectal anastomoses are performed mechanically using circular stapling devices. Despite the introduction of compression anastomosis devices, their usage experience is quite restricted, and no clear advantages over manual or conventional stapled anastomoses have been demonstrated [9, 10].

At this moment, two-row manual circular staplers are the most commonly used devices when performing colorectal anastomosis. Their efficacy and safety results are supported by thousands of procedures performed annually worldwide. Although new devices for performing colorectal anastomosis have appeared in recent years [11, 12], there is no study that demonstrates with a sufficient degree of evidence their superiority to the current MCS.

Medical engineering, in quest of a safer stapled anastomosis, has identified two potential solutions to the issue of anastomotic leakage: a powered circular stapler (PCS) and a three-row manual circular stapler (TRCS). The innovations provided by the PCS have been numerous: firstly, the development of 3D stapling technology designed to evenly distribute compression throughout the anastomosis; secondly, gripping surface technology (GST) that ensures a more delicate handling of tissues; thirdly, this circular stapler delivers enhanced stability, minimizing distal tip movement during triggering; and finally, its controlled tissue compression and adjustable staple height technology that allows the delivery of the desired compression necessary for a strong anastomosis and effective perfusion [13]. In the case of the TRCS, a new design for the stapling process has been chosen, termed “Tri-Staple”™ Technology. This innovative technology for circular staplers consists of three rows of staples (B-shaped staples) at varying heights. The height of the staples increases from the portion closest to the intestinal lumen up to the third row, aiming to achieve the tightest closure possible with minimal disruption of the microvascularization of the anastomotic site [14–16].

The results reported in studies on the reduction of anastomotic complications of two-row manual circular staplers (MCS) and PCS or TRCS [11, 12, 17–30] are controversial, with low evidence of the superiority of PCS or TRCS over traditional two-row manual circular staplers.

The primary objective of this study would be to assess whether PCS and TCRS in colorectal anastomosis demonstrate a lower incidence of anastomotic leaks than two-row manual circular staplers. A secondary goal would be to determine whether the choice of the stapling device has an influence on the risk of post-anastomotic bleeding.

## Material and methods

This meta-analysis was conducted following the updated PRISMA guidelines [31] for systematic reviews and meta-analyses.

### Search strategy

An extensive search of the electronic bibliographic reference databases PubMed, Embase and SCOPUS was performed. The terms used in the identification of articles for meta-analysis were “powered circular stapler”, “circular powered stapler”, “circular” and “powered” and “stapler”, “Echelon” and “circular” and “stapler”, “echelon” and “powered” and “circular” and “stapler”, “three row OR triple” and “circular” “stapler”. No language restrictions were applied. No time limits were set between search criteria.

### Eligibility criteria

The selection criteria were as follows: comparative studies of the results of use between PCS and MCS published in any language in indexed journals, conference abstracts published in indexed journals and papers in which the primary endpoint of the study, anastomotic dehiscence, and/or the secondary endpoint, anastomotic bleeding, were clearly identified and defined. In the case of abstracts, the authors were contacted to obtain all relevant information on the study to be included in this meta-analysis.

Exclusion criteria included studies that did not meet the inclusion criteria, studies in which the actual number of anastomotic leaks was not specified and animal experimental studies.

### Selection of studies and data extraction

Two authors (VPM and JMA) independently searched the three bibliographic databases used. Each author then carried out a selection of relevant studies based on the PICO’s eligibility criteria [32]: the study population comprised patients aged > 18 years who underwent circular stapler colorectal anastomosis, the intervention included the use of novel circular staplers (PCS and TRCS), a comparison between each novel circular stapler and MCS was performed, and the main outcome was anastomotic leak while the secondary outcome evaluated was anastomotic bleeding.

Studies that met the selection criteria were then assessed by title, abstract and full text review. The summary of each study was recorded on a specially designed meta-analysis form using Excel 2016. Variables recorded included the following: authors, year of publication, number of PCS leaks, number of cases without PCS leaks, total number of PCS cases, number of MCS leaks, number of cases without MCS leaks, total number of MCS cases and pathologies included in the study (mixed or malignant exclusively).

After the selection of studies, the two authors compared their results for the final selection of publications. In case of doubt or discrepancy, another author (DMV) was consulted to resolve the differences detected.

### Risk of bias and quality assessment

Two authors (JMA and VPM) independently assessed all papers using the ROBINS I tool. A third author (DMV) confirmed the final determination after discussion.

The overall quality of evidence was addressed using the GRADE methodology [33–36]. The GRADE assessment tool classifies the overall quality of evidence or outcome as high, moderate, low or very low. An outcome may be downgraded one level of certainty for serious problems or two levels for very serious problems, based on the risk of assessment bias, inconsistency, imprecision, indirect evidence or high probability of publication bias. In addition, a GRADE table summarizing the study was performed.

### Assessment of risk of publication bias

Publication bias is the systematic tendency for certain types of studies, typically those with statistically significant or positive results, to be more likely published than studies with non-significant or negative findings. This bias can distort the overall evidence base, as it may not accurately represent the true distribution of research outcomes. It often arises from selective publication by researchers, journal editors, or publishers, and it can lead to an overestimation of treatment effects in meta-analyses, compromising the validity and generalizability of the synthesized evidence. Detection and correction methods, such as funnel plots and statistical tests, are employed to assess and address the impact of publication bias in meta-analytic studies.

Publication biases were visualised by performing funnel plots and assessed by Egger’s test.

The possibility of phacking was also studied in this work. Phacking is defined as the manipulation or inappropriate adjustment of data for the purpose of achieving statistical significance. In the field of meta-analysis, its presence implies the possibility of bias in the results, compromising the statistical validity and reliability of the research. To assess the risk of publication bias and phacking, specific analyses were performed, including the right-skewness test and the flatness test. These tests provide a robust assessment of the possible presence of bias in the results.

### Statistical analysis

The association between the occurrence of anastomotic complications, leakage or bleeding, was assessed by calculating the odds ratio (OR) with its corresponding 95% confidence interval (95%-CI) as summary statistics. This rate expresses the odds of the occurrence of an event in the PCS group compared to the MCS group. In order to better portray the difference between the results of the both circular staplers in the study, the difference in the risk rate of leakage and anastomotic bleeding recorded in the studies was also calculated.

The Mantel–Haenszel method was employed to merge the odds ratios (ORs) for the relevant outcomes, utilizing a random-effects meta-analytical approach. Statistical heterogeneity among the studies was assessed using the Chi-square test, considering *p* value < 0.1 or *I*^2^ > 50% as statistically significant. Inter-study statistical heterogeneity was assessed using Cochrane’s *Q* test and Higgins’ *I*^2^ statistic. In the present meta-analysis, the utilization of the random-effects model was deemed appropriate due to the inherent heterogeneity within the included studies. The patient populations under investigation exhibited diverse characteristics, including variations in surgical techniques, demographics, and clinical conditions, reflecting a global representation. Additionally, the diverse geographical origins of the studies and the inherent variability in surgeon expertise contribute to a broader spectrum of potential effect sizes. The random-effects model accounts for both within-study and between-study variability, offering a more conservative and generalizable estimation of the overall treatment effect. This approach acknowledges and accommodates the anticipated heterogeneity, providing a robust synthesis of evidence that aligns with the inherent diversity of the patient cohorts and surgical practices across the included studies. For the purpose of exploring possible sources of heterogeneity, subgroup analysis and sensitivity analysis were also carried out.

All statistical analyses were performed with RStudio software with R (version 4.3.0) with dmetar, meta and metafor libraries. The *p* value calculated was two-tailed, and if *p* ≤ 0.05, it was considered statistically significant.

## Results

### Selection and description of studies

#### Powered circular stapler

**Fig. 1:**
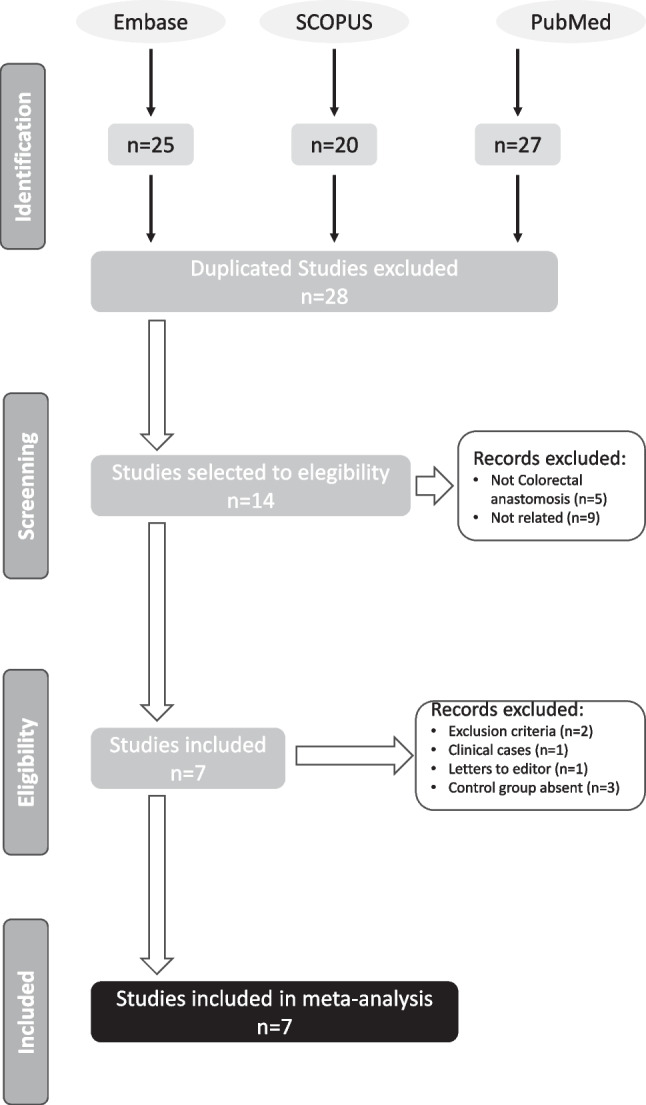
Search in the Embase, PubMed and SCOPUS bibliographic databases yielded a total of 28 articles

The search in the Embase, PubMed and SCOPUS bibliographic databases yielded a total of 28 articles (Fig. 1). Five studies were initially excluded because they dealt with esophagogastric anastomosis, and nine other papers were excluded because they were technical articles on the functioning of the PCS device or were not related to the objectives of this study.

The comprehensive review of the 14 selected papers led to the exclusion of two economic studies because the rate of dehiscence presented did not correspond to a specific group of patients, but was an estimate. One case report, one letter to the editor reporting the initial experience with the use of the PCS device and three cases were also excluded because they were analyses of the experience with the use of PCS without comparison with a control group. Finally, seven papers met the inclusion criteria and were selected for meta-analysis.

The characteristics of the studies included in the meta-analysis (Table 1) exposed that all studies were retrospective observational studies in which case selection for each group (PCS and MCS) was by propensity score matching. Three studies included both benign and malignant pathology while four studies exclusively comprised colorectal cancer. The approaches used were laparoscopic, open or robotic.

**Table 1:** The characteristics of the studies included in the meta-analysis

	**Year**	**Type of study**	**Method**	**Number of cases (after matching)**		**Diagnosis**	**Surgical approach**	**Anastomotic leak**		**Anastomotic bleed**	
								**MCS**	**PCS**	**MCS**	**PCS**
Pla V et al	2021	Retrospective	PSM	179		Mixed	Laparoscopic and open	14 (11.76%)	1 (1.67%)	-	-
				MCS: 119	PCS: 60						
Sylla P et al	2022	Retrospective	PSM	1513		Mixed	Laparoscopic and open	93 (6.9%)	3 (1.82%)	124 (9.2%)	3 (1.82%)
				MCS: 1348	PCS: 165						
Nanishi K et al	2022	Retrospective	PSM	271		CCR	Robotic	11 (8.87%)	10 (6.8%)	1 (0.81%)	1 (0.68%)
				MCS: 124	PCS: 147						
Gonzalez de Julian S et al	2022	Retrospective	PSM	330		Mixed	Laparoscopic and open	22 (13.33%)	8 (4.85%)	-	-
				MCS: 165	PCS: 165						
Shibutani M et al	2023	Retrospective	PSM	126		CCR	Laparoscopic, open and robotic	9 (14.29%)	2 (3.17%)	0	0
				MCS: 63)	PCS: 63						
Vignaly A et al	2023	Retrospective	PSM	290		Mixed	Laparoscopic and open	13 (8.97%)	8 (5.52%)	8 (5.52%)	1 (0.69%)
				MCS: 145	PCS: 145						
Matuhashi N et al	2023	Retrospective	PSM	238		CCR	Laparoscopic	11 (7.91%)	3 (3.03%)	-	-
				MCS: 139	PCS: 99						

**Fig. 2:**
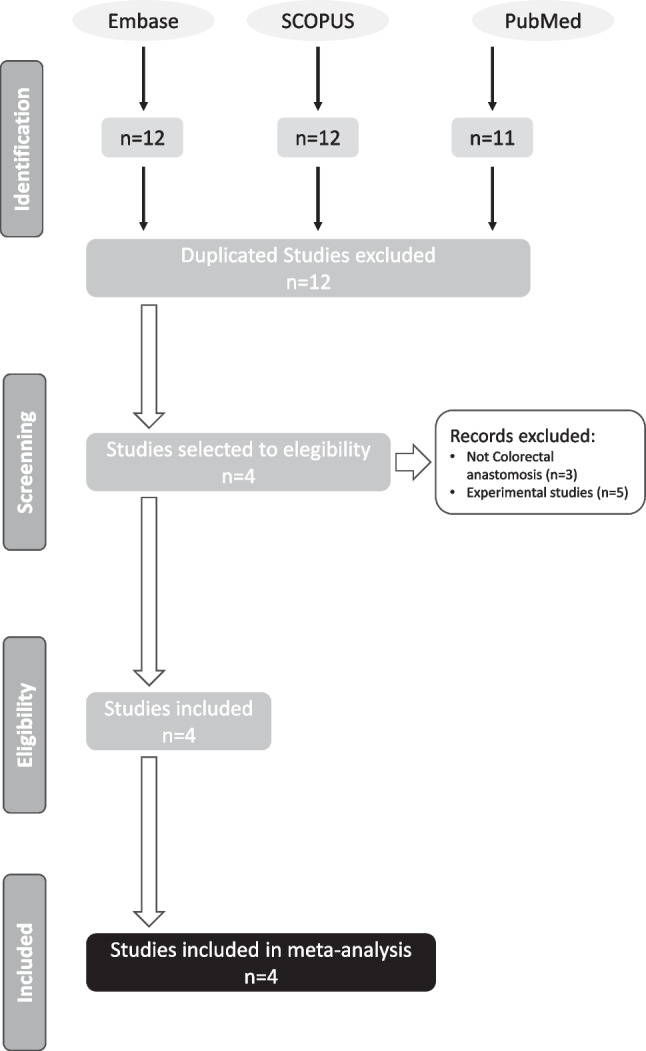
The results of the parameterized search in the selected electronic bibliographic databases initially yielded a total of 12 articles

**Fig. 3:**
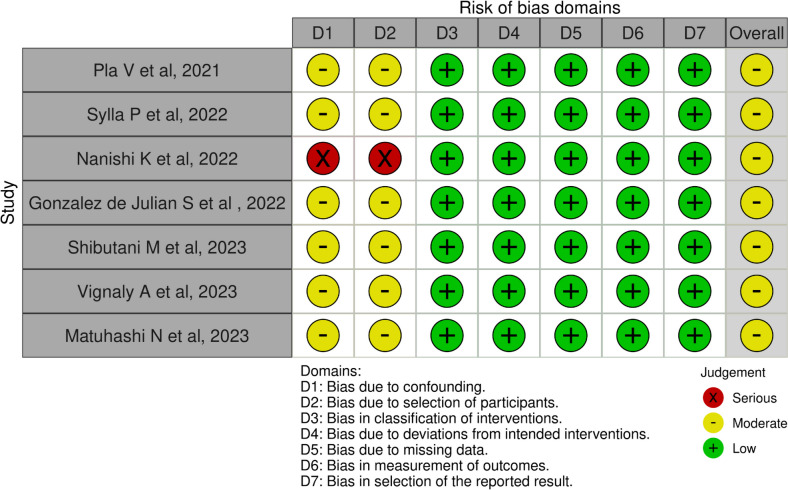
A risk of bias assessment suggested a moderate risk of bias due to confounding factors and the selection of patients in six studies and a serious risk of bias in one

A risk of bias assessment suggested a moderate risk of bias due to confounding factors and the selection of patients in six studies and a serious risk of bias in one (Figs. 2 and 3). The remaining dominions had a low risk of bias.

#### Three-row circular stapler

The results of the parameterized search in the selected electronic bibliographic databases initially yielded a total of 12 articles (Fig. 2). Five studies were excluded as they were experimental studies, and in the remaining three cases, they were not related to the objectives of this study.

The characteristics of the studies included in the meta-analysis for TRCS (Table 2) revealed, similar to PCS, that all cases were retrospective studies. In three of them, propensity score matching was performed, while in the remaining one, matching was based on weights. The diagnoses encompassed both benign and malignant pathologies in three studies, with one study exclusively focused on rectal neoplasms. The surgical approaches included open, laparoscopic, and robotic procedures.

**Table 2:** The characteristics of the studies included in the meta-analysis for TRCS

	**Year**	**Type of study**	**Method**	**Number of cases (after matching)**		**Diagnosis**	**Surgical approach**	**Anastomotic leak**		**Anastomotic bleed**	
								**MCS**	**TRCS**	**MCS**	**TRCS**
Mazaqui J et al.	2022	Retrospective	PSM	68		Mixed	Open and MIS	13 (11.61%)	1 (1.79%)	-	-
				MCS: 112	TRCS: 56						
Quero G et al.	2022	Retrospective	Obs	375		CCR	Laparoscopic, open and robotic	19 (9.64%)	6 (3.37%)	-	-
				MCS: 197	TRCS: 178						
Catarci M et al.	2023	Retrospective	PSM	850		Mixed	Laparoscopic, open and robotic	26 (6.12%)	9 (2.12%)	-	-
				MCS: 425	TRCS: 425						
Wang T et al.	2023	Retrospective	Obs	17,062		Mixed	Laparoscopic, open and robotic	1450 (9.2%)	107 (8.19%)	-	-
				MCS: 15,756	TRCS: 1306						

**Fig. 4:**
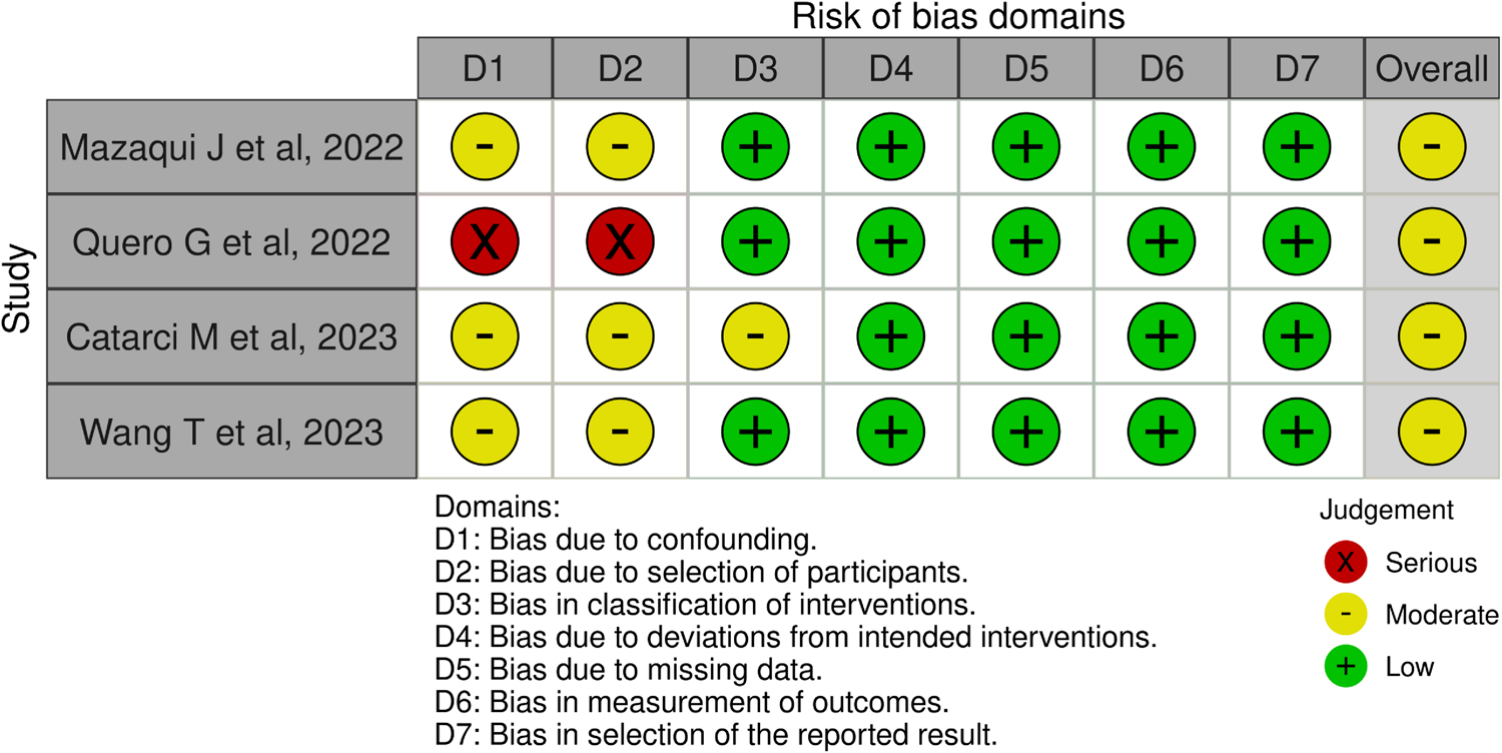
A risk of bias analysis suggested a moderate risk of bias due to confounding factors and selection of patients in three studies and a serious risk of bias in one

A risk of bias analysis suggested a moderate risk of bias due to confounding factors and selection of patients in three studies and a serious risk of bias in one (Fig. 4). One study presented a moderate risk of bias regarding classification of interventions. The remaining dominions had a low risk of bias.

### Estimation of risk of anastomotic leakage

#### Powered circular stapler

The total number of patients included in the meta-analysis was 2947. The distribution by group showed that the most commonly used device was the MCS (MCS: 2103 vs PCS: 844 cases).

**Fig. 5:**
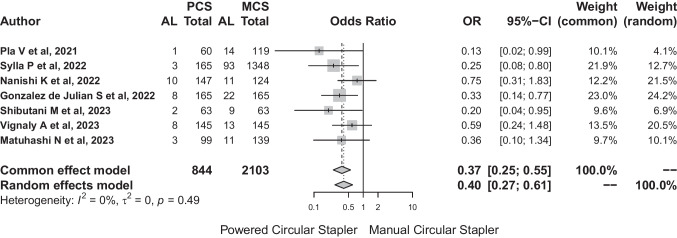
The results of the heterogeneity tests confirmed the consistency of the results (*Q*: 5.44, *p* = 0.489)

Among all the selected studies, 208 cases with anastomotic leakage were noted. Anastomotic leak affected 35 (4.1%) patients in the PCS group versus 173 (8.23%) in the MCS group. A statistically significant association was identified between circular stapling devices (PCS and MCS) and the risk of anastomotic dehiscence (*p* < 0.001). The common-effect model estimated an odds ratio (OR) of 0.3695 (95%-CI: 0.247–0.553). For the random-effects model, the OR was 0.402 (0.266–0.608). Heterogeneity assessment revealed an *I*^2^ index of 0.0%, with variability (*H*^2^: 0.91). The quantitative heterogeneity analysis showed a *τ*^2^ = 0. The results of the heterogeneity tests confirmed the consistency of the results (*Q*: 5.44, *p* = 0.489) (Fig. 5).

The random effects model presented an OR: 0.4 (95% CI: 0.27–0.61). These results consistently suggest a protective effect of PCS relative to AL.

**Fig. 6:**
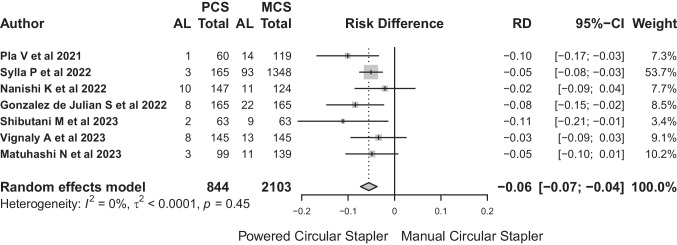
This result suggests a significantly decreased risk of PCS use in relation to anastomotic leakage

The risk difference between the PCS and MCS groups for the random effects model was − 0.06 (95% CI: − 0.07 to − 0.04). This result suggests a significantly decreased risk of PCS use in relation to anastomotic leakage (Fig. 6). The number of patients needed to treat with PCS to avoid leakage would be 17.

**Fig. 7:**
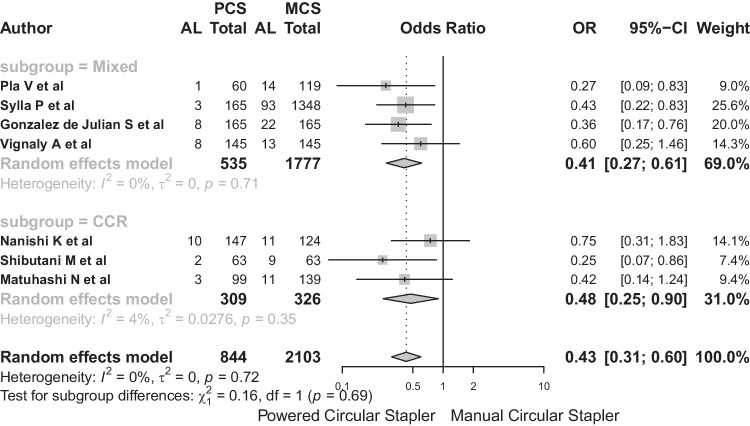
The OR of PCS for LA in the mixed subgroup was 0.41 (95% CI: 0.27–0.61), and in the CRC subgroup, the values obtained were OR: 0.48 (95% CI: 0.25–0.9)

Subgroup analysis, according to diagnostic categories (“Mixed” and “CRC”), showed no substantial differences between these subgroups (Q: 0.16, *p* = 0.693). The OR of PCS for LA in the mixed subgroup was 0.41 (95% CI: 0.27–0.61), and in the CRC subgroup, the values obtained were OR: 0.48 (95% CI: 0.25–0.9) (Fig. 7).

**Fig. 8:**
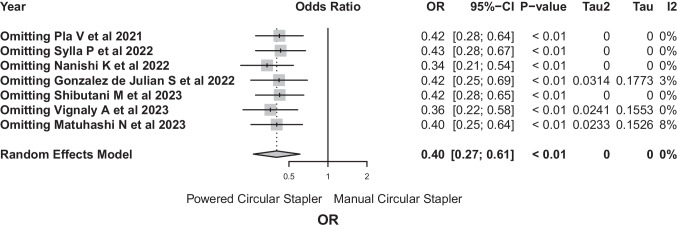
The results showed minimal changes in the effect estimates when individual studies were excluded, which would confirm the robustness and consistency of the overall conclusions of the meta-analysis

A sensitivity analysis was conducted to assess the influence of individual studies on the results of the meta-analysis with fixed effects. The results showed minimal changes in the effect estimates when individual studies were excluded, which would confirm the robustness and consistency of the overall conclusions of the meta-analysis (Fig. 8).

The outcomes of this meta-analysis resulted powered circular stapler resulted certainty high, and it proved to result in a reduction in anastomotic leak (Table 3).

**Table 3:** The outcomes of this meta-analysis

Component						Description							
**P (Patients)**						Patients > 18 years with colorectal anastomosis made with circular stapler							
**I (Intervention)**						Novel circular stapling devices							
**C (Comparison)**						Classic two-row manual stapler vs new circular staplers							
**O (Outcomes)**						Main: postoperative anastomotic leak Secondary: postoperative anastomotic bleed							

**Evolution of certainty**								**No of patients**		**Effect**		**Certainty**	**Importance**
**No. of studies**	**Design of the study**	**Bias risk**	**Inconsistency**	**Indirect evidence**	**Imprecision**		**Other considerations**	**Powered circular stapler**	**Two-row circular stapler**	**Relative (95% CI)**	**Absolute (95% CI)**		
***Powered circular stapler vs two-row stapler***													
**Anastomotic leak**													
7	Observational studies^a^	Not serious	Not serious	Not serious	Not serious		Any	35/844 (4.1%)	173/2103 (8.2%)	**OR 0.40** (0.27–0.61)	**48 less per 1000** (from 59 to 30 minus	⨁⨁⨁⨁ High	Important
**Anastomotic bleed**													
4	Observational studies ^c^	Not serious	Not serious	Not serious	Not serious		Any	5/520 (1.0%)	133/1680 (7.9%)	**OR 0.20** (0.08–0.52)	**62 less per 1000** (from 72 to 36 minus)	⨁⨁⨁⨁ High	Important
***Three-row circular stapler vs two-row circular stapler***													
**Anastomotic Leak**													
4	Observational studies^e^	Serious^f^	Not serious	Not serious	Not serious		Publication bias is strongly suspected All possible residual confounding factors could reduce the demonstrated effect^g,h^	123/1965 (6.3%)	1508/16490 (9.1%)	**OR 0.45** (0.22 a 0.92)	**48 less per 1000** (from 70 to 7 minus)	⨁⨁⨁◯ Moderate	Important

*CI* confidence interval, *OR* odds ratio

^a^Propensity Score Matching was performed to create comparable groups

^b^One study only included rectal cancer and low colorectal anastomosis

^c^Propensity Score Marching was performed to create comparable groups

^d^Ultralow anastomosis in one study

^e^Propensity Score Matching was performed only in one study

^f^Data in two studies originated from two national registries. In one study, the powered circular stapler was considered to have the same results as the two-row circular stapler and was included in this group

^g^Bias publication was suggested in performed statistical tests

^h^High heterogeneity was detected (*I*^2^: 74%, *τ*^2^: 0.0008, *p* = 0.01)

#### Three-row circular stapler

The number of patients included in this group was 18,455 (TRCS: 1965 (10.65%) vs MCS: 16,490 (89.35%)). Anastomotic leakage affected 123 anastomoses in TRCS (6.26%, range: 1.8–8.19%) and 1508 anastomotic leaks were observed in MCS (9.14%, range: 6.12–11.6%

**Fig. 9:**
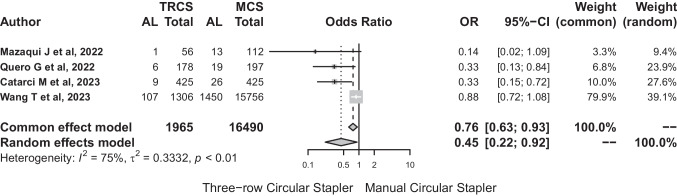
The test of heterogeneity was statistically significant (*Q* = 12.05, *p* = 0.007). The estimated between-study variance (*τ*.^2^) was 0.3332

As for the outcome of meta-analysis in the TRCS group, the random effects model produced an odds ratio (OR) of 0.446 (95%-CI: 0.217 to 0.916). The analysis indicated significant heterogeneity (*I*^2^ = 75.1%). The test of heterogeneity was statistically significant (*Q* = 12.05, *p* = 0.007). The estimated between-study variance (*τ*^2^) was 0.3332 (Fig. 9). These results highlighted the importance of careful interpretation due to observed heterogeneity.

**Fig. 10:**
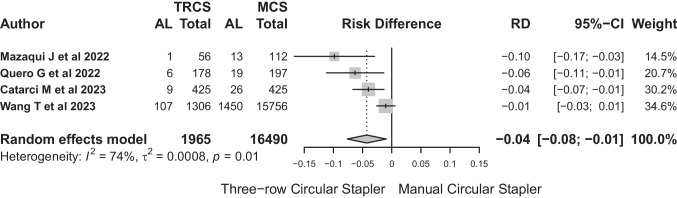
This result suggests a significantly decreased risk of TRCS use in relation to anastomotic leakage

Risk difference between TRCS and MCS was − 0.04 (95%-CI: − 0.04 to − 0.08). This result suggests a significantly decreased risk of TRCS use in relation to anastomotic leakage (Fig. 10). The number of patients needed to treat with TRCS to avoid leakage would be 24.

**Fig. 11:**
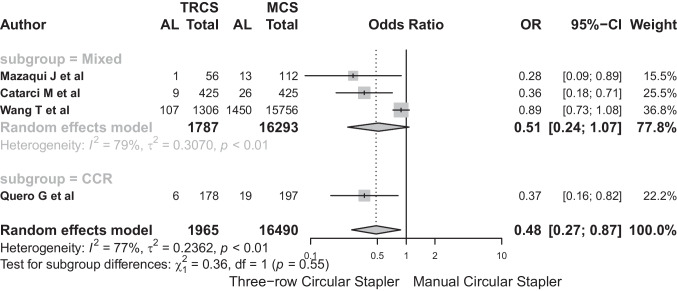
No significant differences were observed between the two subgroups in the subgroup analysis (*Q* = 0.36, *p* = 0.549)

The subgroup analysis identified two subgroups: Mixed (*n* = 3) and CCR (*n* = 1). In the mixed subgroup, the odds ratio (OR) was 0.512 (95%-CI: 0.244 to 1.071), indicating no significant effect, but with notable heterogeneity (*I*^2^ = 78.8%). Conversely, the CCR subgroup demonstrated a significant OR of 0.366 (95%-CI: 0.163 to 0.824). No significant differences were observed between the two subgroups in the subgroup analysis (*Q* = 0.36, *p* = 0.549) (Fig. 11).

**Fig. 12:**
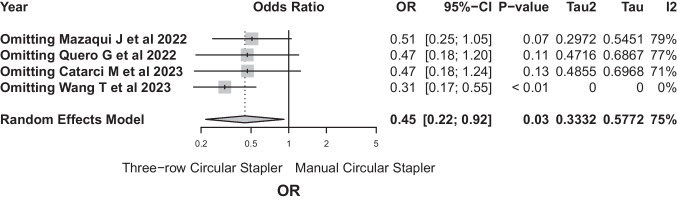
After excluding Wang’s paper, the sensitivity analysis revealed notable alterations in the pooled estimate (OR 0.446, 95%-CI: 0.217–0.916, *p*-value = 0.028)

After excluding Wang’s paper, the sensitivity analysis revealed notable alterations in the pooled estimate (OR 0.446, 95%-CI: 0.217–0.916, *p*-value = 0.028) (Fig. 12). This underscores the influential role of Wang’s study in contributing to observed heterogeneity (*I*^2^ = 75.1%, *τ*^2^ = 0.3332), requiring careful consideration of its influence on the meta-analysis results.

The outcomes of this meta-analysis resulted three-row circular stapler resulted certainty moderate, and it was likely to reduce anastomotic leakage (Table 3).

### Estimation of the risk of anastomotic bleeding

#### Powered circular stapler

Regarding bleeding originating at the anastomosis, only four studies reported this complication. However, one study did not report any cases of anastomotic bleeding.

The total number of patients included in the analysis was 2200 with 138 (6.27%) postoperative anastomotic bleeds. A statistically significant relationship (*p* < 0.001) was confirmed between the occurrence of postoperative anastomotic bleeding and both circular staplers.

**Fig. 13:**
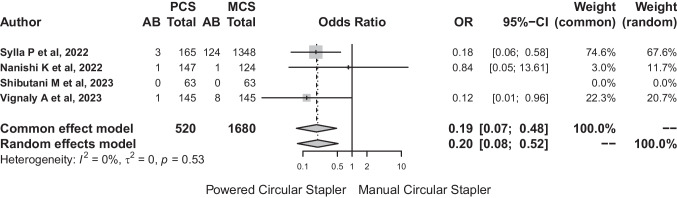
The heterogeneity test showed no significant difference between studies (*Q*: 1.29, *p* = 0.526)

Anastomotic bleeding was identified in five patients (0.96%) in the PCS group versus 133 (7.91%) in the MCS group. For anastomotic bleeding, the common effect and the random effects model showed similar results. The common-effect model showed an OR for AB for bleeding of 0.19 (95% CI: 0.07–0.048) while the random-effects model showed an OR for AB for this complication of 0.2 (95% CI: 0.08–0.52). Low heterogeneity was observed according to the *I*^2^. The heterogeneity test showed no significant difference between studies (*Q*: 1.29, *p* = 0.526) (Fig. 13).

**Fig. 14:**
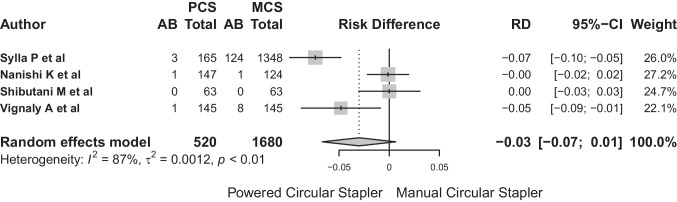
The risk difference was − 0.03 (95% CI: − 0.07 to − 0.01), so the number of PCS needed to avoid bleeding would be 34 cases

The risk difference for postoperative anastomotic bleeding between PCS and MCS based on the random-effects model showed that the model had an *I*^2^: 87%. The risk difference was − 0.03 (95% CI: − 0.07 to − 0.01), so the number of PCS needed to avoid bleeding would be 34 cases (Fig. 14).

**Fig. 15:**
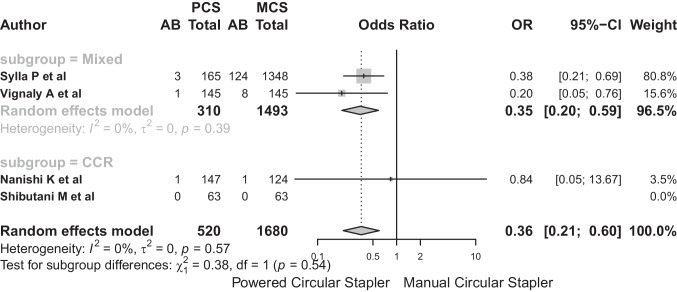
The OR for CS in AB was 0.35 (95%-CI: 0.2–0.59) in the mixed subgroup and 0.36 (95%-CI: 0.21–0.6) in the CRC subgroup

Subgroup analysis, in the case of anastomotic bleeding based on diagnostic categories (“mixed” and “CCR”), showed no significant differences between these subgroups (*Q*: 0.38, *p* = 0.538) although the model showed significant heterogeneity with *I*^2^: 87%. The OR for CS in AB was 0.35 (95%-CI: 0.2–0.59) in the mixed subgroup and 0.36 (95%-CI: 0.21–0.6) in the CRC subgroup (Fig. 15).

**Fig. 16:**
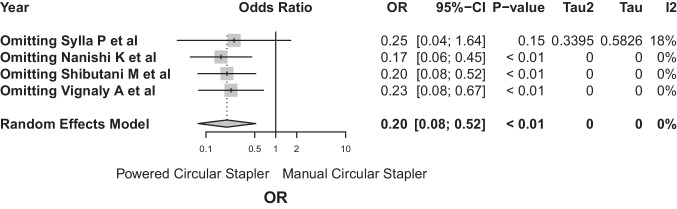
Sensitivity analysis in the case of anastomotic bleeds showed minimal changes in effect estimates when excluding each individual study, supporting the robustness and consistency of the overall conclusions of the meta-analysis

Sensitivity analysis in the case of anastomotic bleeds showed minimal changes in effect estimates when excluding each individual study, supporting the robustness and consistency of the overall conclusions of the meta-analysis (Fig. 16).

Powered circular stapler resulted in a large reduction in anastomotic bleed.

#### Three-row circular stapler

As for the TCRS, none of the articles documented this postoperative anastomotic complication.

### Assessment of publication bias

#### Powered circular stapler

##### Anastomotic leakage

**Fig. 17:**
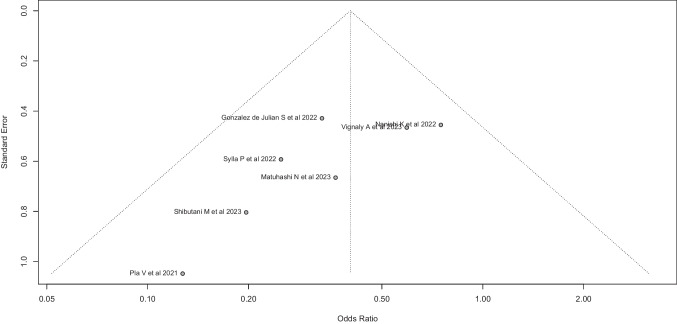
Egger’s test ruled out the existence of a significant asymmetry in the dispersion funnel (constant: − 2.329 (95%-CI: (− 4.32 to − 0.33), *p* = 0.071)

The funnel plot of the random effects model showed a symmetrical distribution of studies. Egger’s test ruled out the existence of a significant asymmetry in the dispersion funnel (constant: − 2.329 (95%-CI: (− 4.32 to − 0.33), *p* = 0.071) (Fig. 17).

The results of the right-skewness test (*p* = 0.842) and flatness test (*p* = 0.022) suggest that there is no evidence of significant skewness or flatness in the *p* values of the included studies. These findings indicate a low probability of phacking practices in the meta-analysis, supporting the validity of the meta-analysis results.

##### Anastomotic bleeding

**Fig. 18:**
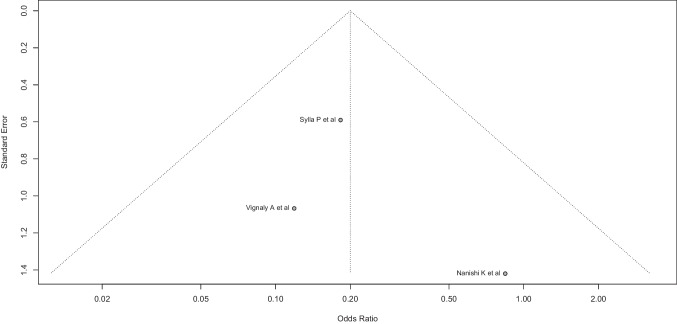
As to anastomotic bleeding, the random effects model funnel plot and Egger’s test showed no evidence of significant asymmetry in the dispersion funnel (constant: 0.988 (CI-95%: − 4.04 to 4.04, *p* = 0.64)

As to anastomotic bleeding, the random effects model funnel plot and Egger’s test showed no evidence of significant asymmetry in the dispersion funnel (constant: 0.988 (CI-95%: − 4.04 to 4.04, *p* = 0.64) (Fig. 18).

However, due to the limited number of cases, neither the right-skewness test nor the flatness test could be calculated in the anastomotic bleeding setting.

#### Three-row circular stapler

**Fig. 19:**
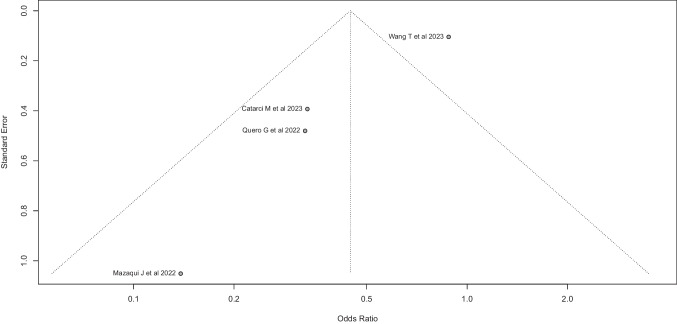
Egger’s analysis indicated an intercept significantly different from zero (*k* =  − 2.532, 95%-CI: − 3.37 to − 1.69, *p* = 0.027)

In the evaluation of possible biases in the case of the TCRS meta-analysis, evidence of asymmetry in the funnel plot was observed. Egger’s analysis indicated an intercept significantly different from zero (*k* =  − 2.532, 95%-CI: − 3.37 to − 1.69, *p* = 0.027) (Fig. 19), suggesting the presence of asymmetry.

## Discussion

Experience with new circular staplers (TRCS and PCS) is limited. Both circular staplers lack prospective randomized trials comparing their results with MCS devices. Existing studies on the use of PCS or TRCS primarily consist of small series reporting experimental original articles, initial experiences or retrospective observational studies comparing the new devices with MCS.

PCS and TRCS represent two different concepts on how to improve the results of a colorectal anastomosis. Given the available evidence, it remains challenging to definitively determine which of these new circular staplers, along with their respective technical solutions, yield superior results.

The decrease in the incidence of anastomotic leakage reported by some publications on the experience of the new PCS [13, 19, 25, 27, 37] and TRCS [12, 29, 38] compared with the two-row circular staplers should be viewed with caution because they are observational studies, not randomized clinical trials. Furthermore, there is some heterogeneity in the studies because multicentre studies have been included, some of them with cases from large national databases or on-going clinical trials not designed to assess the difference in leakage rates among different devices [29, 39, 40]. At this point, it may be premature to state that MCS devices have worse outcomes than the newer PCS or TRCS devices primarily because there is no clear evidence in this regard [22, 24, 30].

Our outcomes have attempted to provide some degree of evidence for a possible decrease in the risk of anastomotic complications in colorectal anastomosis with PCS and TRCS in human clinical practice. To our knowledge, this is the first meta-analysis in which an attempt has been performed to assess the possible relationship between new circular staplers and MCS on anastomotic leakage and bleeding.

Ideally, meta-analyses should include prospective randomised trials; however, it is common practice to use retrospective studies. This meta-analysis used retrospective studies in which propensity score matching was applied in order to make the study groups fully comparable. For some authors, although with limitations, studies using matching techniques could demonstrate causality, as is the case with prospective randomised trials, even with limited sample sizes [41–43].

The publications included in the study are likely to reflect current standard practice, as they include studies from different countries around the world, involving benign and malignant pathology, along with open and minimally invasive (laparoscopic and robotic) approaches (Tables 1 and 2).

The results of this meta-analysis, although they should be viewed with caution, showed that the use of both PCS and TRCS in colorectal anastomoses has a protective effect on anastomotic complications. PCS demonstrated a 63.05% risk reduction in anastomotic leakage, with an odds ratio (OR) of 2.71 (95%-CI: 1.81–4.05) for achieving a successful anastomosis. In comparison, TRCS exhibited a 55.4% risk reduction in anastomotic leakage, with a calculated OR of 2.24 (95%-CI: 1.09–4.71) for achieving a satisfactory anastomosis compared to MCS. Regarding anastomotic bleeding, PCS reduced the risk of bleeding by 80% or alternatively expressed; the risk of bleeding was five times lower with this device than with MCS. Calculation of the risk difference rate for leakage and for anastomotic bleeding showed that the use of PCS could prevent one leak after 17 anastomoses performed and TRCS could prevent one leak after 25 anastomosis, which would support data reported in previous studies [29]. The calculation of the number of PCS applications needed to prevent bleeding would be 20.

Anastomotic leakage risk reduction when using PCS and TRCS in performing colorectal anastomoses appears to be similar, with a slight advantage for PCS. However, these results should be approached cautiously due to the type of studies included and the high heterogeneity observed in the TRCS meta-analysis. In the case of the PCS meta-analysis, a low heterogeneity of the studies should be highlighted.

Sensitivity analysis did not detect significant problems in the PCS meta-analysis. However, in the case of TRCS, the work presented by Wang et al. [30] was the one that introduced the greatest heterogeneity to the analysis, probably because sample size and the values recorded for leakage differed from those provided by the other three studies [12, 28, 29].

The diagnosis of the pathology motivating surgical intervention in this meta-analysis included benign and malignant pathology in three studies [17, 24, 37] in the PCS meta-analysis, similar to that of the TRCS meta-analysis [12, 29, 30]. However, subgroups analysis, according to pathology, did not seem to have an influence on the results and confirmed the validity and robustness of both meta-analyses.

The outcome of the study of possible publication biases has been intriguing. In relation to PCS meta-analysis, neither the funnel plot nor Egger’s test detected publication bias. Moreover, phacking problems were also ruled out, which would give robustness and validity to the results of this analysis. In turn, in the case of the TRCS meta-analysis, a significant asymmetry was observed in the funnel plot, which was confirmed by Egger’s test.

The possible causes of the asymmetry detected in the case of the TRCS meta-analysis could be bias publication and the small number of studies available, which were also highly heterogeneous. Consequently, the results of this meta-analysis should be interpreted with forethought.

The phacking study could only be performed in the case of the PCS meta-analysis for anastomotic leakage, ruling out its existence. In the remaining cases, due to the characteristics of the studies included in the analysis, it could not be calculated.

The manufacturer of TRCS reports an 80% decrease in the risk of leakage, 140% greater perfusion allowed into the staple line and reduction in firing compared to PCS [44] on its website. These magnificent and surprising results are supported by internal company reports and experimental studies in dogs primarily [45, 46]. Results of this meta-analysis do not directly compare the PCS with the TRCS. Nevertheless, the comparison of PCS and TRCS vs MCS has shown a greater decrease in the risk of anastomotic leak with PCS. As a consequence, it is unlikely that TRCS will ever decrease the risk of anastomotic leak when compared to PCS, as suggested by the manufacturer [44], at least in human clinical practice.

In our opinion, the reduction of the risk of anastomotic complications obtained by PCS is probably the main effect of the new concept of three-dimensional design of the staples and the gripping surface technology mainly due to the reduction of compressive forces in anastomotic tissues improving healing conditions. Although to a lesser extent, but also of note, the powered firing process of the device provides more stability with a reduction of sharing forces in the anastomotic site. Also, some studies suggest that the ease of use of the PCS would drastically reduce involuntary movements during firing and therefore possible damage to the anastomosis. A circular stapler is not a magic bullet that will create an anastomosis irrespective of the surgeon’s skills. However, the application of PCS by decreasing the sharing forces at the site of the anastomosis could make the tightness of the closure easier to achieve, as suggested by some authors [22, 47]. Therefore, we believe that PCS should not be included in the manual two-row stapled devices in the same way that three-row staplers should not be categorized in the group of manual circular staplers. Although we have not been able to find any studies comparing PCS with TRCS, the study by Catarci et al. [29], when comparing TRCS versus MCS, includes PCS (9.41%) anecdotally in MCS group. This could have induced biases in the results as the anastomotic leak rate between PCS and MCS appears to be significantly different.

One of the limitations of this meta-analysis is the small number of studies available and the absence of randomised clinical trials. All the studies included in the meta-analyses, except Quero et al. and Want et al. studies [30, 38], have included propensity score matching, with the inherent limitations of this statistical method. The validity and reality of results of two of the included studies [37, 39] has been questioned by other authors who appreciated possible caveats in the propensity score matching [29], although they did not question the results of other studies with similar characteristics [12]. The conclusions drawn from this study should be viewed mindfully interpreted. Obviously, prospective randomised studies are needed to answer the question of which would be the best circular stapling device.

Although the findings of this study warrant careful consideration due to the previously explained reasons, this study is the first meta-analysis aiming to provide evidence on the risk of postoperative anastomotic leakage and bleeding in colorectal anastomoses, comparing the novel circular stapling devices with conventional circular staplers. The subgroup and sensitivity analyses provided robustness and validity to the results. Furthermore, a meticulous evaluation of the risk of publication bias and phacking has been undertaken to enhance the methodological rigour of this investigation.

While it would be tempting to make an indirect comparison of the results of PCS and TRCS with the studies included in this report, it would be methodologically incorrect. The certainty of evidence was high for the PCS vs MCS meta-analysis while it was only moderate for the TRCS vs MCS meta-analysis. In the case of anastomotic bleed, certainty was high too. The outcomes of this meta-analysis should be confirmed by conducting randomized clinical trials in which the comparison of the three available circular staplers: two-row, three-row and powered circular stapler should be considered.

In conclusion, the results of this meta-analysis suggest that a powered circular stapler could have a significantly lower risk of leakage and anastomotic bleeding than a two-row manual circular stapler. The three-row circular stapler may also have a reduced risk of anastomotic leakage compared with the two-row manual circular stapler. The reduction of the risk of anastomotic leakage could be greater in the powered circular stapler group than in the three-row circular stapler group. Finally, prospective randomized trials are needed to confirm the findings obtained in this study.

## Data Availability

The search in the electronic bibliographic databases (PubMed, SCOPUS and Embase) was carried out through the servers of the University of Valencia, which also provided the articles for subsequent analysis. The authors of the study by González de Julián S et al. were contacted by e-mail to obtain all the data necessary for the study to be included in the meta-analysis. The protocol for the search and preparation of the meta-analysis can be provided upon request to the authors.
